# Lectin Pathway Enzyme MASP-2 and Downstream Complement Activation in COVID-19

**DOI:** 10.1159/000525508

**Published:** 2022-07-11

**Authors:** Maximilian Peter Götz, Mikkel-Ole Skjoedt, Rafael Bayarri-Olmos, Cecilie Bo Hansen, Laura Pérez-Alós, Ida Jarlhelt, Thomas Benfield, Anne Rosbjerg, Peter Garred

**Affiliations:** ^a^Laboratory of Molecular Medicine, Department of Clinical Immunology, Section 7631, Copenhagen University Hospital − Rigshospitalet, Copenhagen, Denmark; ^b^Department of Immunology and Microbiology, Faculty of Health and Medical Sciences, University of Copenhagen, Copenhagen, Denmark; ^c^Department of Infectious Diseases, Copenhagen University Hospital − Amager and Hvidovre, Hvidovre, Denmark; ^d^Department of Clinical Medicine, Faculty of Health and Medical Sciences, University of Copenhagen, Copenhagen, Denmark

**Keywords:** Mannose-binding lectin-associated serine protease 2, MASP-2, Assay, COVID-19, Inflammation, Outcome

## Abstract

Mannose-binding lectin-associated serine protease 2 (MASP-2) is the main activator of the lectin complement pathway and has been suggested to be involved in the pathophysiology of coronavirus disease 2019 (COVID-19). To study a possible association between MASP-2 and COVID-19, we aimed at developing a sensitive and reliable MASP-2 ELISA. From an array of novel mouse-monoclonal antibodies using recombinant MASP-2 as antigen, two clones were selected to create a sandwich ELISA. Plasma samples were obtained from 216 healthy controls, 347 convalescent COVID-19 patients, and 147 prospectively followed COVID-19 patients. The assay was specific towards MASP-2 and did not recognize the truncated *MASP2* splice variant MAP-2 (MAp19). The limit of quantification was shown to be 0.1 ng/mL. MASP-2 concentration was found to be stable after multiple freeze-thaw cycles. In healthy controls, the mean MASP-2 concentration was 524 ng/mL (95% CI: 496.5–551.6). No significant difference was found in the MASP-2 concentrations between COVID-19 convalescent samples and controls. However, a significant increase was observed in prospectively followed COVID-19 patients (mean: 834 ng/mL [95% CI: 765.3–902.7, *p* < 0.0001]). In these patients, MASP-2 concentration correlated significantly with the concentrations of the terminal complement complex (ρ = 0.3596, *p* < 0.0001), with the lectin pathway pattern recognition molecules ficolin-2 (ρ = 0.2906, *p* = 0.0004) and ficolin-3 (ρ = 0.3952, *p* < 0.0001) and with C-reactive protein (ρ = 0.3292, *p* = 0.0002). Overall, we developed a specific quantitative MASP-2 sandwich ELISA. MASP-2 correlated with complement activation and inflammatory markers in COVID-19 patients, underscoring a possible role of MASP-2 in COVID-19 pathophysiology.

## Introduction

The complement system is an important part of innate immunity and is divided into three main pathways: the classical, alternative, and lectin pathway. The lectin pathway initiates the complement cascade upon pattern recognition molecule (PRM) binding to pathogen- or damage-associated molecular patterns. When in circulation, PRMs of the lectin pathway (i.e., mannose-binding lectin [MBL], collectin-11 [CL-11], ficolin-1, −2, and −3) can be found in complex with the MBL-, ficolin-, and collectin-associated serine proteases (MASPs) MASP-1, MASP-2, and MASP-3 and the truncated versions, termed MBL-, ficolin-, and collectin-associated proteins (MAPs) MAP-1 (Map44) and MAP-2 (Map19) [1].

The 5 MASPs and MAPs are derived from two different genes termed *MASP1* (MASP-1, MASP-3, and MAP-1) and *MASP2* (MASP-2 and MAP-2) by alternative splicing [2]. The structure of the MASPs and MAPs is very similar. The MASPs are composed of a heavy/alpha chain and a light/beta chain. The heavy/alpha chain, starting from the N-terminus, is comprised of two CUB domains (C1r/s, embryonic sea urchin protein, bone morphogenetic protein) separated by an EGF (epidermal growth factor) segment and followed by two complement control protein (CCP) domains. The heavy chain is responsible for the calcium-dependent interaction with the collagen-like region of the PRMs. The light/beta chain comprises the serine protease domain containing a cleavage site that separates the heavy and light chains upon MASP activation. The two chains are then held together by an interchain cysteine bridge. The truncated, nonenzymatic MAP-2 contains the first CUB and the EGF domain, while MAP-1 carries most of the heavy chain except for the last CCP. MASP-1, −3, and MAP-1 contain 4, 7, and 2 N-linked glycans, respectively, whereas MASP-2 and MAP-2 are not glycosylated [2, 3, 4, 5].

Upon binding to pathogen- or damage-associated molecular patterns, MASP-2 initiates the complement cascade by cleaving C4 into C4a, C4b, while both MASP-1 and MASP-2 cleave C2 into C2a and C2b. It has also been shown that MASP-2 might cleave C3 directly and amplify the alternative pathway loop [6]. MASP-2 is the main activator of the lectin pathway and has been found to be 1,000 times more catalytically active than its classical pathway counterpart C1s [2, 7]. In addition, it has been shown that MASP-1 can convert zymogen MASP-2 to active MASP-2 [8, 9].

MASP-3 is crucial for the cleavage of profactor D to its active form (i.e., factor D), enabling activation of the alternative complement pathway [7]. MAP-1, and possibly MAP-2, may function as regulators of complement activation [10, 11, 12]. The MASPs and MAPs are found in the circulation as homodimers [2, 7, 10, 13]. Apart from the canonical complement substrates, both MASP-1 and MASP-2 have been shown to cleave and activate coagulation factors such as prothrombin and fibrinogen [13, 14, 15, 16, 17].

The study of MASP-2 in health and disease is left behind due to the limited number of available reliable assays. MASP-2 has been suggested to be a driving force in several inflammatory diseases such as IgA nephropathy and bone marrow transplantation thrombotic microangiopathies [6, 18, 19]. Elevated or decreased levels of MASP-2 in serum and plasma have been associated with multiple diseases, and MASP-2 has been suggested as a diagnostic or prognostic marker in inflammatory diseases [20, 21, 22, 23, 24]. Recently, several lines of evidence indicate that MASP-2 might be involved in the pathophysiology of COVID-19 [13, 25, 26, 27].

The disease course of COVID-19 can range from asymptomatic over mild symptoms to respiratory failure and ultimately death in the most severe cases, hence, new and additional clinical markers might be a valuable indicator for the progression of the disease and a useful tool for the choice of treatment [28, 29, 30, 31, 32]. Thus, to further address the possible role of MASP-2 in COVID-19 and other diseases, we generated and characterized a panel of mouse monoclonal antibodies (mAbs) against human MASP-2 and established a reliable and sensitive MASP-2 sandwich ELISA.

## Materials and Methods

### Buffers

The following buffers have been used, all buffers are based on distilled water: PBS (10.1 mM Na_2_HPO_4_, 1.5 mM KH_2_PO_4_, 137 mM NaCl, 2.7 mM KCl), PBS-Tw (PBS, 0.5% Tween-20), PBS-Tw-EDTA (PBS-Tw, 20 mM EDTA), sample buffer (PBS-Tw, 20 mM EDTA, 0.5% vol/vol bovine serum, 6.67 nM polyclonal mouse IgG isotype control [Thermo Fischer Scientific, Waltham, MA, USA]), elution buffer (100 mM glycine, pH 2.7), protein G binding buffer (20 mM NaH_2_PO_4_, pH 7.0).

### Production of Recombinant Protein

rMASP-2 (NCBI RefSeq: NM_006610.4), with a C-terminal hexahistidine-tag and a Ser613Ala substitution, was produced using the Gibco ExpiCHO^TM^ expression system (Thermo Fischer Scientific) in serum-free media according to the manufacturers' instructions. The His-tagged rMASP-2 supernatant was then aliquoted for single-use and stored at −20°C.

### Generation and Selection of Anti-MASP-2 mAbs (A-MASP-2 mAbs)

Outbred RPMI mice were immunized three times via subcutaneous injection. Immunization was performed with 20 μg of purified rMASP-2 diluted 1:1 with Freund's incomplete adjuvant (Sigma-Aldrich, St. Louis, MO, USA). Responding mice were injected with an intravenous dose of 20 μg rMASP-2 three days before the fusion. The fusion and the following selection of clones were done by hybridoma technology [33, 34] using SP2 myeloma cells (in-house). Positive clones were screened with 0.5 μg/mL rMASP-2 (in-house) as antigen-coated directly on MaxiSorp microtiter plates (Thermo Fischer Scientific). To ensure specificity of the clones, they were further screened against recombinant MAP-2 (rMAp19), normal human serum from healthy donors, and MBL-deficient serum. The supernatant of the clones was expected not to react with the truncated MASP-2 splice variant MAP-2 and MBL-deficient serum. Another positive control besides rMASP-2 was an MBL-MASP-2-complex (produced analogously to Valdimarsson et al. [35]).

Selected mAbs were purified, via affinity chromatography using an ÄKTA Pure system (GE Healthcare, Chicago, IL, USA) with a HiTrap Protein G column (GE Healthcare). The column was equilibrated using Protein G Binding Buffer (in-house) until a stable UV baseline was reached. The supernatant of the selected clones, diluted 1:1.5 in Protein G Binding Buffer, was applied to the column. Bound antibodies were eluted with elution buffer in fractions of 2 mL. The column was washed with Protein G Binding Buffer and stored in 20% (vol/vol) ethanol. Fractions containing the most antibody were pooled and dialyzed against PBS. For the ELISA setup, mAbs were biotinylated, using 20% (wt/vol) (+)-Biotin N-hydroxysuccinimide ester (Sigma-Aldrich), for 3 h at room temperature (RT), and later dialyzed against PBS to remove excess biotin.

To select the best fitting pair of coating and detection mAbs in a sandwich ELISA setup, all selected and purified mAbs were screened against each other in every combination. Each detection mAb was biotinylated. Serum and rMASP-2 in a constant concentration were used for screening as the analyte. The pair with the highest intensity, and no increased background was then selected as the final coating and detection mAbs.

A two-dimensional dilution of both mAbs was used to assess the optimal concentrations of coating and detection antibodies in a sandwich ELISA setup. Serum, plasma, and rMASP-2 at constant concentrations have been used as analytes. The individual combination of concentrations was judged based on the background signal, as well as the signal intensity. Analogous, varying incubation times and different sample buffers have been assessed on the same measurements.

### Serum Pull-Down

To immunoprecipitate MASP-2 from serum, 3 μg of mAbs F3-5 and F3-100 and a purified mouse IgG1κ isotype control (BD Biosciences, Franklin Lakes, NJ, USA) were separately incubated with end-over-end shaking for 1 h at RT with 50 μL of Dynabeads sheep anti-mouse IgG (Invitrogen, Waltham, MA, USA) each. The coupled beads were washed with PBS-Tw three times and afterward, the beads were incubated for 1 h at RT with a serum pool diluted 1:1 in PBS-Tw-EDTA. Beads were washed again with PBS-Tw three times, and the bound proteins were eluted by heating the beads in NuPAGE® LDS Sample Buffer (Invitrogen). The eluates were applied on an SDS-PAGE as described below. Another mAb generated as described, F3-35, was biotinylated and used as a primary antibody on the final Western blot membrane as F3-35 did show a strong signal intensity when used as a primary antibody compared to other generated a-MASP-2 mAbs.

### SDS-PAGE and Western Blot

Before application to the NuPAGE® Tris-Acetate 4–12% gel (Invitrogen), all samples were denatured by the addition of NuPAGE® LDS Sample Buffer (Invitrogen), followed by heating to 95°C for 5 min. Samples were reduced by adding NuPAGE® Sample Reducing Agent (Invitrogen). The loaded gel was run in an electrophoresis cell (Bio-Rad, Hercules, CA, USA) coupled to an electrophoresis power supply (Amersham Biosciences, Amersham, UK) at 150 V and max. 300 mA for 75 min. The gel was stained with InstantBlue Coomassie Protein Stain (Abcam, Cambridge, UK) or blotted onto a nitrocellulose membrane (Invitrogen) at 30 V and max. 300 mA for 75 min. Membranes were blocked with 5% (wt/vol) skim milk powder (Merck Millipore, Burlington, MA, USA) in PBS-Tw and incubated overnight (o/n) with 0.5 μg/mL primary antibody. The membranes were afterwards incubated for 1 h at RT with rabbit anti-mouse HRP P0260 (Agilent Technologies, Santa Clara, CA, USA) in a 1:5,000 dilution or with streptavidin conjugated to HRP (Cytiva, Marlborough, MA, USA) in a 1:2,000 dilution. Before being developed, using the SuperSignal^TM^ West Femto Maximum Sensitivity Substrate (Thermo Fisher Scientific), membranes were washed again in PBS-Tw. Membranes were then recorded using the Odyssey® Fc imaging system (LI-COR Biosciences, Lincoln, NE, USA).

### MASP-2 Specific Sandwich ELISA

One of the two selected antibodies, a-MASP-2 mAb F3-5, was coated on MaxiSorp microtiter plates with 2 μg/mL in PBS o/n at 4°C. Plates were washed three times with PBS-Tw after each step. Incubation of plates took place at RT on a shaker, unless otherwise stated. In each well, 100 μL of a sample (serum, plasma, etc.) and the calibrator in 2-fold dilutions in sample buffer were added to the plate and incubated for 1 h. A sample buffer with high EDTA concentration was used to disassociate MASP-2 from PRMs so that the individual proteins could be measured [10]. Afterward, biotinylated a-MASP-2 mAb F3-100 was applied to the plate at 1.5 μg/mL in PBS-Tw and incubated for 1 h. Streptavidin-HRP conjugate (RPN1231V; Sigma-Aldrich) in a 1:2,000 dilution in PBS-Tw was added and incubated for 1 h. For color development, TMB One (Kem-En-Tec Diagnostics, Taastrup, Denmark) was used as a substrate. After 15 min, the reaction was stopped using 0.3 H_2_SO_4_. OD was measured at 450 nm using a Synergy HT microplate reader (BioTek, Winooski, VT, USA).

### Assay Validation: Parallelism, Precision, Lower Limit of Detection, Working Range, Dynamic Range, Variation, and Spike-In/Recovery

This assay was validated following various recommendations [36, 37], similar to already well-established ELISA assays [38, 39]. An EDTA plasma pool (described below) was used for calibration. This plasma pool was set to contain 400 ng/mL MASP-2 [9, 24, 38, 40, 41, 42, 43]. For assessing parallelism and recovery, supernatant from ExpiCHO cells producing rMASP-2 was used. It was added to the mentioned ELISA assay setup in a 2-fold 12-point dilution to obtain a reference standard curve running from approx. 80 ng/mL (1:5 diluted plasma) to 0.4 ng/mL (1:10,240 diluted plasma).

To assess parallelism of the assays' different samples, duplicates of a 12-point dilution of rMASP-2 supernatant, plasma, serum, and calibrator were measured. The logarithmically transformed dilution curves were then fit to a nonlinear log(agonist) versus response equation (variable slope, 4PL), and the resulting hill slopes were then compared.

Another validation, the spike-in and recovery test, describes the assays' ability to measure the complete MASP-2 content in each sample. For this, an EDTA plasma pool was spiked with 20 or 50% (vol/vol) or rMASP-2 CHO supernatant. The spiked samples, as well as the non-spiked ones, were measured and interpolated to the EDTA pool calibrator. Then, the theoretical concentrations of the spiked samples and the measured concentrations can be set into a ratio to calculate the recovery percentage.

The linearity of dilutions on a plate was calculated by interpolating each concentration of an 11-point dilution curve duplicate of the calibrator to itself. These calculated concentrations for each dilution duplicate were then divided by the average interpolated concentration of the calibrator curve. This was done on 25 separate plates for 25 dilution rows in duplicates. The dilution linearity was considered acceptable if more than 80% of points for each dilution were within a 20% deviation from the mean concentration.

The assays' precision was measured by dividing the OD signal by the average OD signal of each duplicate of an 11-point dilution curve. The working range of the assay was set to the range of dilutions in which the variation coefficient (CV) of both the assays' precision and its linearity was below 20%.

The lower limit of quantification (LLOQ) was calculated by averaging the OD of the blank duplicates on a plate and adding three times its standard deviation to it. This value was then interpolated to nanogram/milliliter using the standard curve on each plate. The final LLOQ was determined by averaging every plate's LLOQ.

Additionally, inter- and intra-assay variations were calculated. Inter-assay variation was estimated by measuring the same plasma pool in duplicates on 25 plates over 7 days, interpolating their respective MASP-2 concentration, and calculating the resulting CV. Intra-assay variation was calculated by measuring the same plasma pool in 35 duplicates (70 measurements) on the same plate, interpolating their concentration, and again calculating their CV.

Lastly, to validate the assays' ability to pick up most MASP-2 in solution, a spike-in and recovery test was performed. For this, a plasma pool was spiked with 10% of rMASP-2 supernatant. These samples' concentrations were then interpolated, compared, and checked for plausibility to the interpolated concentrations of the plasma pool and the supernatant separately.

### Stability of rMASP-2 and MASP-2 in Serum and Plasma Samples

For insights into the stability of MASP-2 in serum and plasma samples, as well as rMASP-2 supernatant produced in-house, serum and plasma pools from ten healthy donors and rMASP-2 supernatant were stored at different temperatures. Samples have been stored at 4°C, RT, and 37°C for 24, 48, and 72 h. Additionally, to evaluate stability after freezing and thawing, samples were frozen at −80°C and thawed to RT up to four times. Concentrations of MASP-2 in these sample duplicates were interpolated individually and compared to pretreatment levels. All samples were interpolated to an EDTA plasma pool with only one freeze and thaw cycle set to 400 ng/mL MASP-2 [9, 24, 38, 40, 41, 42, 43]. The concentration of each dilution row was averaged on the plausible interpolated concentrations of every dilution in the working range of the assay. Different time points of samples were compared by a two-way ANOVA without correction always to time point 0 or one freeze-thaw cycle, respectively.

### EDTA-Plasma Pool Used for Standardization

A small pool of EDTA plasma of healthy donors (*n* = 10) was used to calibrate the concentration of MASP-2 in the assay. Plasma samples were obtained by 30 min of incubation of freshly drawn blood samples in EDTA tubes and subsequent centrifugation for 10 min at 1,000× *g*. A pool of EDTA plasma from healthy donors was created to function as a reliable and stable standard for all assays. For this, the supernatant after centrifugation was pooled and stored at −80°C in 1 mL aliquots. The EDTA plasma pool was set to a concentration of 400 ng/mL MASP-2 [9, 24, 38, 40, 41, 42, 43].

### Cohort of Hospitalized Patients with COVID-19

MASP-2 in EDTA plasma was measured in a cohort of adults admitted to the Copenhagen University Hospital − Amager and Hvidovre, with a confirmed severe acute respiratory syndrome coronavirus 2 (SARS-CoV-2) infection. All were admitted to the hospital between March 10th and May 31st of 2020. This cohort has been characterized previously [32]. 188 samples of this cohort were measured with the above-described assay. 147 of those measured samples were successfully linked to a patient each on the first days of their admission. Statistics on those 147 patients can be seen in Table 1. Various information about the patients and their clinical status was linked to the samples and accessible for this study. Survival was evaluated by 30-day mortality, meaning a 30-day follow-up on patients' survival. All samples were stored at −80°C.

### Convalescent Individuals Previously Infected with SARS-CoV-2

EDTA plasma of 350 convalescent individuals, previously infected with SARS-CoV-2, registered by the Copenhagen University Hospital − Herlev, was collected [44]. The cohort was grouped into five stages of severity of their COVID-19 disease (asymptomatic, mild, moderate, severe, and critical). Additional information like the age and gender of the patient was available. All available samples were measured for their MASP-2 levels. 347 samples were interpolated, and their concentration was linked to the available clinical characteristics of the individual samples. All plasma samples were stored at −80°C.

### Anonymous Healthy Control Cohort

A control cohort was established by collecting EDTA plasma from 217 blood donors from May 2017 to February 2019, before the COVID-19 pandemic. These were used as a reference control for normal MASP-2 levels. These samples were anonymized, and no additional information was available. 216 samples were able to be interpolated. All samples were stored at −80°C.

### Statistical Analyses

For statistical analyses, GraphPad Prism version 9.2.0 (RRID: SCR_002798; GraphPad Software, San Diego, CA, USA) and R version 4.1.0 (RRID: SCR_001905; R Foundation for Statistical Computing, Vienna, Austria) were used. Unless otherwise stated, all measurements were done in duplicates. The concentration of MASP-2 in samples was calculated by fitting a four-parameter logistic curve to the standard and interpolating from this curve by regression analysis. If not mentioned otherwise, concentrations of populations are given as the mean plus its lower and upper 95% confidence interval. Outliers in every given group have been eliminated using an outlier calculator by GraphPad Prism. For correlation studies in COVID-19 patients, the interpolated concentrations of MASP-2 were used on a continuous scale. Normal distribution of each given group of values was tested by an Anderson-Darling test. Normality of stability measurements was confirmed by using a Shapiro-Wilk test due to the small sample size. When needed, log transformations to the base of 2 were used to achieve a normal distribution of values. Correlation of various factors to MASP-2 concentrations was done by one-way ANOVA, Kruskal-Wallis test, unpaired *t* test or Mann-Whitney, or by fitting a linear model without covariates, as appropriate and as normality of the distribution allowed it. When working with noncontinuous variables, a logistic (generalized linear model) and no log transformation was used. Analyses of statistical significances when comparing COVID-19 patient cohorts with one another were done by a Kruskal-Wallis test. When analyzing stability measurements, differences to the initial sample were tested via two-way ANOVA. Differences in nontreated samples of sera and plasma were compared using a Kruskal-Wallis test. The linear trend between the mean of ascending age groups of infected individuals was calculated via an ordinary one-way ANOVA with a test for a linear trend of the nonlogarithmically transformed values. Significance was set to a *p* value below 0.05. Asterisks representing the *p* value can be interpreted as ns, *p* > 0.05; *, *p* ≤ 0.05; **, *p* ≤ 0.01; ***, *p* ≤ 0.001; and ****, *p* ≤ 0.0001.

## Results

### mAb Generation

A total of 17 mAbs were selected based on specific reactivity against recombinant (r) MASP-2 by a direct ELISA. The selected mAbs showed no cross-reactivity towards MAP-2 and no signal development in MBL-deficient and MASP-2-deficient serum (data not shown). All clones were evaluated pairwise in a sandwich ELISA setup. Two clones were selected: F3-5 as the capture antibody and F3-100 biotin-conjugated as the detection antibody, based on their signal-to-noise ratio when measuring MASP-2 in EDTA plasma and serum, rMASP-2 supernatant, and purified MBL/MASP-2 complexes. No background signal could be detected when substituting one of the mAbs with an irrelevant mouse antibody (data not shown). Both antibodies were isotyped as mouse IgG1κ.

### Western Blot and Serum Pull-Down

**Fig. 1:**
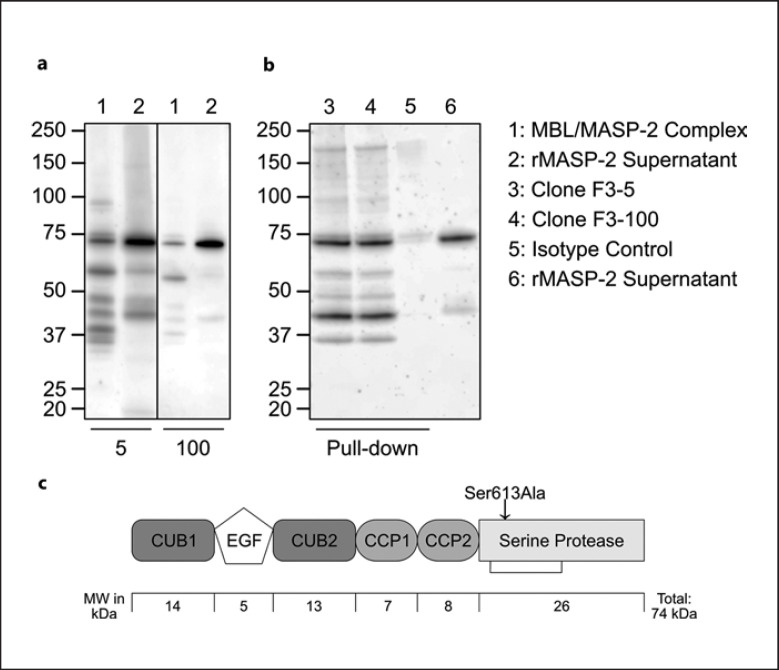
**a** Western blot using biotinylated F3-5 and F3-100 as detection antibodies as indicated. Sample in lane 1: an MBL-MASP-2-complex, lane 2: recombinant MASP-2 culture supernatant. **b** Immunoprecipitation (pull-down) of normal human serum using F3-5, F3-100, and a mouse IgG1κ isotype control, as indicated. Samples were detected using a-MASP-2 mAb F3-35. Recombinant zymogen MASP-2 culture supernatant was added in lane 6 as a positive control. **c** A schematic structure of MASP-2 with approximate molecular weights of each domain. The zymogen form of rMASP-2 was achieved by introducing the mutation shown as Ser613Ala, theoretically minimizing autoactivation. Activated MASP-2 is held together by a disulfide bond in serine protease domain, shown as a connecting line [46, 47].

Figure 1 shows Western blots of clones F3-5 and F3-100 as detection antibodies, as well as an immunoprecipitation of serum MASP-2 (serum pull-down). A purified MBL/MASP-2 complex and rMASP-2 culture supernatant were loaded onto a gel and detected with clones F3-5 and F3-100, as seen in Figure 1a. Both antibodies show similar binding patterns with both samples. Full-size MASP-2 in a no-reduced form weighs about 75 kDa, whereas the heavy chain weighs 48 kDa and the light chain weighs about 26 kDa [17, 45]. When measured in complex with MBL, MASP-2 can be detected in a pattern with a prominent band at slightly under 75 kDa. Other, weaker bands could be detected as well. rMASP-2 supernatant only shows two major bands with both antibodies, the by far most prominent band at just below 75 kDa and one between 40 and 50 kDa. When immunoprecipitating from serum, both F3-5 and F3-100 seem to bind efficiently to endogenous MASP-2 as they both precipitated full-length MASP-2 (∼75 kDa) and the MASP-2 heavy chain (∼45 kDa) (Fig. 1b, lane 3 and 4). Culture supernatant containing rMASP-2, used as a positive control, shared the most prominent bands with the immunoprecipitated samples. Comparing the latter with the MBL/MASP-2 complex detected by clones F3-100 or F3-5, the pattern and most bands seem to be identical. No significant bands were visible when precipitating with an IgGκ isotype control antibody (Fig. 1b, lane 5). Similarly, membranes without the primary detection antibody did not show any bands (data not shown). A schematic structure of MASP-2 with approximate molecular weights of each domain can be seen in Figure 1c. The zymogen form of rMASP-2 was achieved by introducing the mutation shown as Ser613Ala, theoretically minimizing autoactivation. When activated, the serine protease domain of rMASP-2 is held together by a disulfide bond, shown in Figure 1c [46, 47].

### Assay Validation

Based on previous studies [9, 24, 38, 40, 41, 42, 43], MASP-2 concentrations in the current assay were interpolated using an EDTA plasma pool set to 400 ng/mL as a calibrator. The calibrator was fit with a four-parameter log(antagonist) versus response curve with an *R*^*2*^ above 0.995 for all curves.

**Fig. 2:**
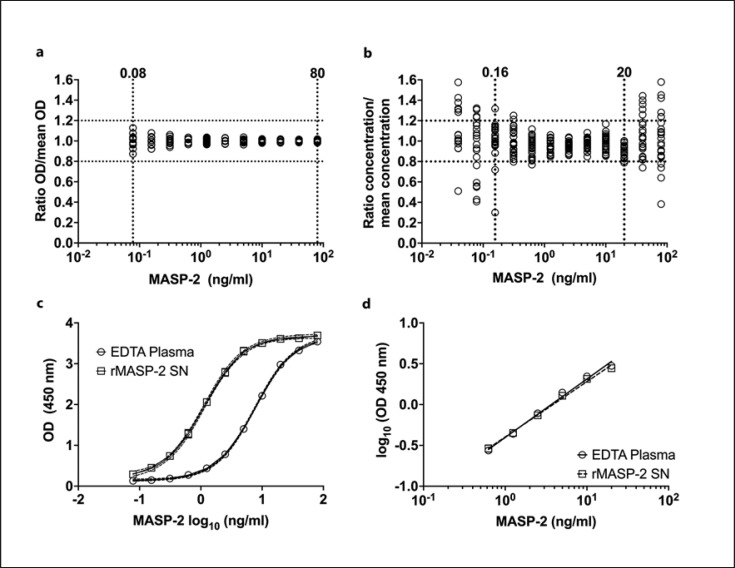
**a** Precision over an 11-point dilution curve. **b** Dilution linearity measured on 25 plates over a 12-point dilution curve. **c** Dilution curves of rMASP-2 supernatant and EDTA plasma. **d** Linear range for evaluating parallelism between rMASP-2 supernatant and EDTA plasma.

To evaluate precision, the OD values reflecting the MASP-2 level of six different donors in duplicates were measured and divided by the average of those ODs. Every dilution from 20% to 0.02% plasma (approx. 80 ng/mL to 0.08 ng/mL, respectively) showed a CV below 20% (Fig. 2a).

Dilution linearity was found to be satisfactory (i.e., <20% variation on 80% of plates) in the range of 0.156 ng/mL to 20 ng/mL. Forty nanogram/milliliter was calculated to be just out of the range but for most cases, still acceptable (Fig. 2b). As the LLOQ was defined as the mean background signal of one plate plus three times its standard deviation, the limit of 25 plates varied slightly from plate to plate and averaged to 0.12 ng/mL of MASP-2.

To prospectively base the assay on purified rMASP-2 as a calibrator, the parallelism between EDTA plasma and rMASP-2 culture supernatant was calculated. Figure 2c and d show the linear range of the calibrator and rMASP-2 supernatant (0.63–20 ng/mL) after being log-transformed and fitted with a simple linear regression (*R*^*2*^ > 0.99). There was no significant difference between the slopes (0.716 for the calibrator and 0.678 for the rMASP-2 supernatant). Similarly, the hill-slopes of both full dilution ranges fitted with a four-parameter log(antagonist) versus response curve were not significantly different; 1.361 for the calibrator and 1.358 for rMASP-2 supernatant (Fig. 2d). Recovery of rMASP-2 spiked in an EDTA plasma pool sample was above 95% (online suppl. Fig. 1, to be found in the online suppl. material; for all online suppl. material, see www.karger.com/doi/10.1159/000525508).

This intra-assay variation was determined to be 7.5%, while the inter-assay variation was calculated to be 9.3%. Taking all validation measurements and calculations into consideration, the working range of the assay ranges from 0.16 to at least 20 ng/mL.

### Stability

**Fig. 3:**
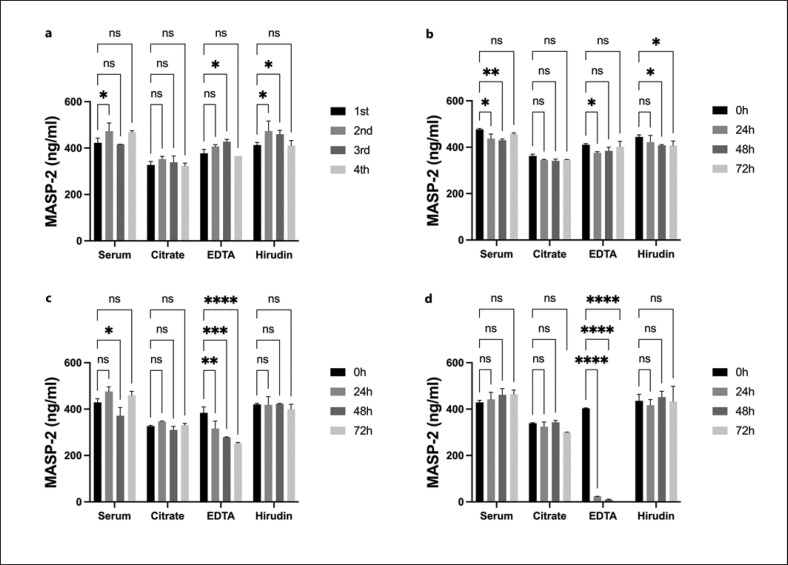
Stability measurement of MASP-2 in serum and plasma. **a** Freeze and thaw cycles. **b** Storage at 4°C. **c** Storage at RT. **d** Storage at 37°C. Significances have been calculated by a two-way ANOVA compared to the first sample of each group. Data are represented as mean and error bars as standard deviations for each time point. ns, *p* > 0.05; *p ≤ 0.05; **p ≤ 0.01; ***p ≤ 0.001; ****p ≤ 0.0001.

Measurement of stability was done for serum and plasma (citrate, EDTA, and hirudin plasma), pooled from the same ten healthy donors (Fig. 3). Stability was measured at 0, 24, 48, and 72 h at 4°C (Fig. 3b), RT (Fig. 3c), or 37°C (Fig. 3d). Additionally, another batch of serum and plasma pools was frozen and thawed for up to four cycles (Fig. 3a).

Concentrations of MASP-2 in serum and plasma seem to be very stable in most conditions. Freeze-thaw cycles did not influence MASP-2 levels (Fig. 3a). Only EDTA plasma showed a stepwise reduction in MASP-2 concentration when plasma was stored at RT for 24–72 h (Fig. 3c) and a significant drop in concentration after storage at 37°C for 24 h (Fig. 3d).

When comparing serum and the three most common plasma preparations, MASP-2 levels seemed to vary slightly between those. On average, citrate plasma was observed to contain the lowest amount of MASP-2 detected, followed by EDTA plasma, hirudin plasma, and serum (mean concentrations: 339, 394, 428, and 439 ng/mL MASP-2, respectively). Concentrations, compared to serum, were significantly lower for citrate plasma (*p* < 0.0001) and EDTA plasma (*p* = 0.02).

### Calibrator EDTA Plasma Pool

The collected plasma pool of 10 individuals was measured multiple times on the assay for standardization. Dilution curves and duplicate measurements showed minimal variation in inter- and intra-assay comparisons of always less than 10% with interpolated concentrations. Intra-assay variation of crude optical density values was repeatedly measured to be below 5%. Using this reliable and stable plasma pool as standard, highly volatile rMASP-2 theoretically only needs to be compared to said standard once and thus reduces uncertainties in measurement and interpolation of concentrations significantly.

### MASP-2 Levels in Normal Controls and COVID-19 Cohorts

**Fig. 4:**
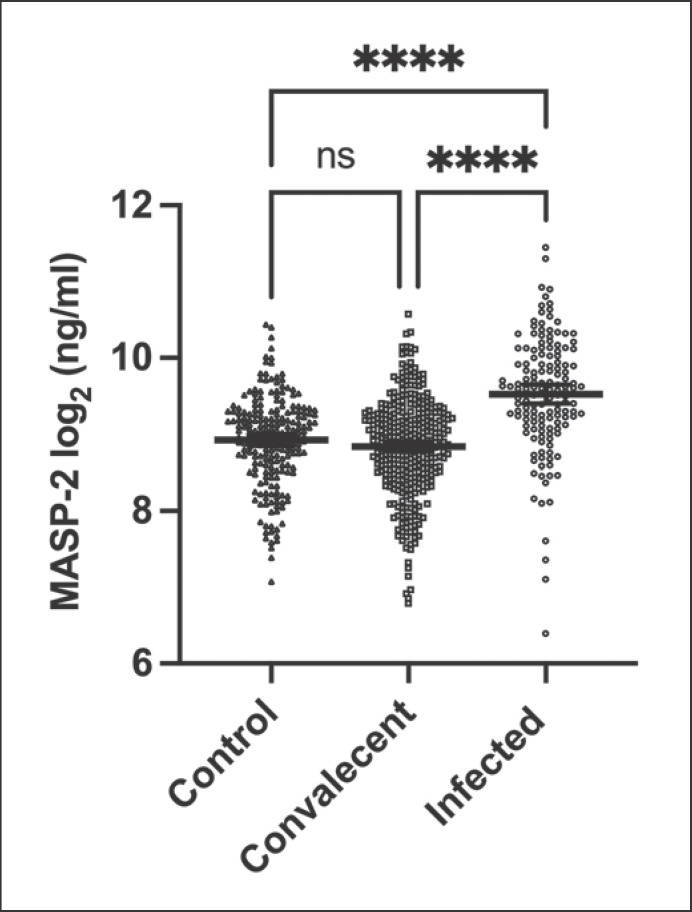
Comparison of MASP-2 levels of prepandemic control cohort, convalescent individuals, and infected patients. Significance in the difference of means calculated via Kruskal-Wallis test of to the base of 2 logarithmically transformed values. Data points represent MASP-2 levels of individuals. Lines and error bars represent mean levels and 95 % CI. ns, *p* > 0.05; *p ≤ 0.05; **p ≤ 0.01; ***p ≤ 0.001; ****p ≤ 0.0001.

The MASP-2 assay was applied to two different COVID-19 cohorts; convalescent individuals and individuals in the acute phase of a SARS-CoV-2 infection at the time of sample collection. Both were compared to a prepandemic control group of blood donors. As seen in Figure 4, there was no significant difference between the mean MASP-2 levels of the convalescent individuals and the controls. On the other hand, mean MASP-2 plasma levels in individuals infected with SARS-CoV-2 at the time of sample collection were significantly elevated compared to all other groups (*p* < 0.0001). Mean values and 95% confidence intervals for the control cohort, convalescent, and infected individuals were measured to be 524 ng/mL (95% confidence interval of the mean [95% CI]: 496.5–551.6), 500.2 ng/mL (95% CI: 477.9–522.5), and 834 ng/mL (95% CI: 765.3–902.7), respectively.

### Anonymous Healthy Control Cohort

Interpolated concentrations of the control cohort showed a very similar distribution of MASP-2 levels and an almost identical mean value to convalescent individuals. Mean values were also not significantly different from the calibrator EDTA pool.

### Convalescent Individuals

**Fig. 5:**
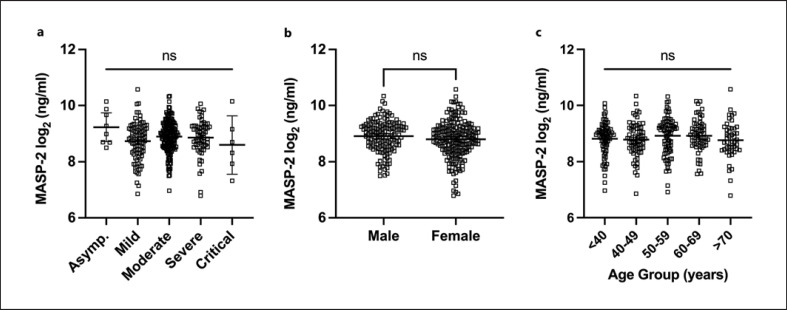
**a–c** Correlation of MASP-2 concentrations within the cohort of convalescent individuals. **a** Mean MASP-2 concentration of convalescent individuals compared by the severity of their originally endured SARS-CoV-2 infection. **b** MASP-2 levels grouped and mean compared by gender. **c** MASP-2 levels grouped and mean compared by age group. Significance in the difference of means calculated via Kruskal-Wallis test (**a, c**) and Mann-Whitney test (**b**) to the base of 2 logarithmically transformed values. Data points represent MASP-2 levels of individuals. Lines and error bars represent mean levels and 95 % CI. ns, *p* > 0.05.

Relevant descriptive factors in this cohort can be found in Table 1. The MASP-2 concentrations in the convalescent individuals were plotted against several factors, like the severity of previous SARS-CoV-2 infection (Fig. 5a), sex (Fig. 5b), and age (Fig. 5c). None of these factors showed a significant correlation to MASP-2 levels.

### Hospitalized Individuals with Moderate to Severe COVID-19

**Fig. 6:**
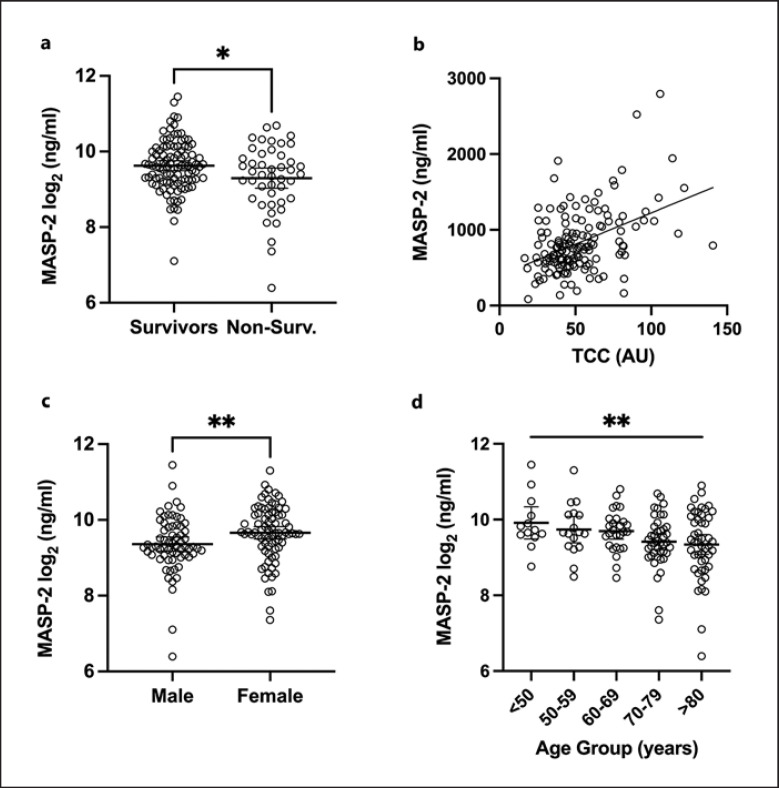
**a–d** Correlations of MASP-2 concentration within the cohort of hospitalized individuals with COVID-19. **a** Mean MASP-2 concentrations of infected individuals grouped and compared by survivor status at 30 days after sample collection. **b** Linear correlation of calculated MASP-2 concentrations and measured TCC values in plasma samples of infected individuals. **c** Difference in mean concentrations of MASP-2 grouped by gender of infected individuals. **d** MASP-2 concentrations and mean concentrations of ascending age groups of infected individuals. Significance in the difference of means calculated via unpaired *t* test (**a**), Mann-Whitney test (**c**), and ordinary one-way ANOVA (**d**) to the base of 2 logarithmically transformed values. **b** Regression was calculated via a simple linear regression of the nonlogarithmically transformed values of MASP-2 and TCC. **d** The aforementioned significant (**) linear trend between means of the ascending age groups was calculated via an ordinary one-way ANOVA with a test for a linear trend of the nonlogarithmically transformed values. **a, c, d** Data points represent MASP-2 levels of individuals. Lines and error bars represent mean levels and 95 % CI. ns, *p* > 0.05; * *p* ≤ 0.05; **p ≤ 0.01; ***p ≤ 0.001; ****p ≤ 0.0001.

A description of the cohort can be found in Table 2. As seen in Figure 6a, individuals with a recorded fatal outcome of their SARS-CoV-2 infection had significantly lower MASP-2 concentrations compared to survivors in the same cohort, 733.7 ng/mL (95% CI: 620.6–847.8) and 878.3 ng/mL (95% CI: 793–963.6), respectively. Survival was assessed for up to 30 days after the sample was taken. Notably, the mean of the lower MASP-2 concentrations of deceased patients was still higher than the mean concentration in the control cohort, 733.7 ng/mL (95% CI: 620.6–847.8) and 524 ng/mL (95% CI: 496.5–551.6). As seen in Table 3, MASP-2 concentrations showed a significant positive correlation with ficolin-2 (ρ = 0.29, *p* = 0.0004) and ficolin-3 (ρ = 0.395, *p* < 0.0001). No significant correlation with the other lectin pathway PRMs was observed (i.e., CL-11, ficolin-1, and MBL). A significant positive correlation was observed with the complement activation product TCC (ρ = 0.36, *p* < 0.0001) (Fig. 6b). A significant correlation was also observed with the inflammatory marker C-reactive protein (ρ = 0.33, *p* = 0.0002). As seen in Figure 6c, women tended to have higher MASP-2 levels than males when infected with SARS-CoV-2, 744.2 ng/mL (95% CI: 643.7–844.7) for men and 907.1 ng/mL (95% CI: 813.1–1,001) for women (*p* = 0.0031). There was a significant decreasing linear trend between all means of ascending age groups (*p* = 0.0042, slope = −77.4 ng/mL MASP-2), suggesting that MASP-2 levels decreased slightly in older infected individuals in this cohort (<50: 1,088 ng/mL [95% CI: 701.9–1,474], 50–59: 950.4 ng/mL [95% CI: 689.7–1,211], 60–69: 887.4 ng/mL [95% CI: 754.1–1,021], 70–79: 754.5 ng/mL [95% CI: 651.6–857.5], >80: 763.5 ng/mL [95% CI: 644.9–882]). When comparing each single age group's mean to one another via one-way ANOVA, no one single mean was significantly different from another (Fig. 6d).

## Discussion

We successfully generated multiple mAbs against rMASP-2. We validated them for specificity by testing against rMASP-2, its truncated splice variant MAP-2 (Map19) [11], as well as MASP-2 binding in MBL-deficient and normal human serum. All clones were able to detect rMASP-2, as well as native serum MASP-2. This was further verified by Western blots of an MBL-MASP-2 complex and rMASP-2 supernatant. The theoretical molecular mass of MASP-2 is about 76 kDa in its nonreduced form. We found it migrated with a slightly lower molecular mass of (approx. 74 kDa), in line with previous observations [10, 48]. Additionally, MAP-2, expected to be around 19 kDa, was not detected. Other weaker bands suggest degradation, aggregation, or activation products of MASP-2. In the immunoprecipitations and regular Western blots, both clones F3-5 and F3-100, used in the assay, bound to a band between 40 and 50 kDa, possibly belonging to the A chain of MASP-2 (expected to be 48 kDa). This means that the epitopes of both clones bind to the A chain, excluding the shared domains of MAP-2. This indicates a binding site on the CUB2, CCP1, or CCP2 domains [16, 17, 48, 49]. Other bands are believed to be degradation products [11].

We used an EDTA plasma pool, from a range of normal donors, assumed to have an approximated concentration of 400 ng/mL MASP-2 based on literature [9, 24, 38, 40, 41, 42, 43]. Using this assumption, we quantified the concentration of MASP-2 in serum, plasma, and rMASP-2. This approach offers a stable and robust measurement with the most comparable calibrator.

The MASP-2 sandwich ELISA was validated for precision, dilution linearity, parallelism, inter- and intra-assay variation, and recovery ability. All together, the obtained data suggest a minimal working range of 0.16 to more than 20 ng/mL MASP-2. The estimated limit of quantification, as well as the working range, together with the stability of the assay and apparent robustness, makes this assay a reliable and promising alternative to current assay setups. Additionally, without relying on, often costly, sometimes poorly described commercially available antibodies, the here-generated mAbs, and the in-house-produced assay might find several applications, especially in complement-related research [11, 21].

The MASP-2 sandwich ELISA was very stable when applied both in serum and plasma under various conditions and after multiple freeze-thaw cycles. The only exception was EDTA plasma, in which MASP-2 possibly degrades when incubated for prolonged times at higher temperatures. Thus, the improper storage of EDTA plasma samples might interfere with the accurate MASP-2 measurement. Freeze and thaw cycles did not affect either serum or plasma MASP-2 levels, irrespectively, suggesting that the assay is robust and reliable and can be used on previously frozen and thawed biobank material. Compared to hirudin plasma and serum, divalent cation chelating agents such as citrate and EDTA did affect the MASP-2 signal. Suggesting that removing divalent cations might create an assay environment that might reduce the MASP-2 signal, which should be considered when comparing results when blood is drawn into collection tubes with or without chelating agents. Regardless, the reason for this discrepancy is not yet solved and will require further investigations, but calcium ions may stabilize the tertiary structure of MASP-2 in a configuration needed for the epitope recognition of the different antibodies applied in this study. Moreover, MASP-2 dimerization and binding to the PRMs are calcium-dependent, which could also influence the binding of antibodies in the assay. Additionally, as seen in the online supplementary material in supplementary Figures 1 and 2, rMASP-2 was partly activated and subsequently measured on the established sandwich ELISA. Overlapping curves and similar interpolated concentrations of activated and nonactivated rMASP-2 suggest no discrimination of the assay between MASP-2 in different activation statuses.

Previous studies have shown that MASP-2 in plasma is normally distributed [38]. We also observed this when testing MASP-2 in a group of pre-COVID-19 plasma samples obtained from Danish blood donors. No difference in distribution or concentration was observed between the blood donors and a group of COVID-19 individuals sampled within 11 weeks after symptom onset [44]. However, in patients admitted to the hospital with COVID-19, a significant elevation of MASP-2 concentration was observed compared with controls. This elevation was in the magnitude of around 1.6 suggesting that MASP-2 may be a weak acute phase reactant, which is compatible with earlier findings [41]. The convalescent individuals had a concentration comparable to controls indicating that the MASP-2 concentration within weeks after an inflammatory stimulus will return to baseline level. MASP-2 levels did as well not differ in the convalescent groups of different disease severity.

Infected hospitalized individuals display significantly higher levels of MASP-2 compared to control or convalescent groups (Fig. 6). This might be due to general inflammation, as hyperinflammation has been reported, especially in patients with severe cases of COVID-19 [50]. The complement system has also been reported to hyperactivate in several pathways, as well as MASP-2 itself has been shown to autoactivate in presence of SARS-CoV-2, leading to complement activation and coagulation [30, 51]. Elevated MASP-2 might aid in that process of complement activation and might act proinflammatory in higher concentrations.

MASP-2 levels in hospitalized individuals with COVID-19 correlated with the inflammation marker C-reactive protein. This contributes to the notion that MASP-2 is an acute phase reactant and that the MASP-2 gene might be induced due to inflammatory stimuli.

MASP-2 levels were also correlated to lectin pathway PRMs ficolin-2 and ficolin-3 and to the generation of TCC. This correlation points to an essential role of MASP-2 in the complement cascade initiated by SARS-CoV-2. Since increased levels of TCC have been associated with disease severity in COVID-19 patients [52], MASP-2 might be considered a possible point of therapeutic intervention as well as a possible prognostic marker.

We observed a slight but significant decrease in MASP-2 concentration in those COVID-19 patients who died during the observation period, which seems to be counterintuitive to the notion that elevated complement activation might be associated with disease severity. Alternatively, this subgroup of most severe cases might exhibit consumption of MASP-2 through activation, as this has also been shown for C3 in COVID-19 [53, 54, 55].

However, the pathophysiology of COVID-19 is complex, and we also observed a slight overall inverse correlation of MASP-2 levels with increasing age and male sex in infected individuals, both associated with fatal outcome in COVID-19 patients [56, 57, 58]. No association between sex and age was seen in convalescent individuals.

Thus, the true impact of MASP-2 on disease severity in COVID-19 requires further studies. In conclusion, we developed a sensitive, specific, and robust sandwich ELISA to quantitate MASP-2. The concentrations obtained by this assay were correlated with complement activation and inflammatory markers in COVID-19 patients. These results underscore a possible role of MASP-2 in COVID-19 pathophysiology.

## Statement of Ethics

All procedures involving the handling of human samples are in accordance with the principles described in the Declaration of Helsinki and ethically approved by the Regional Ethical Committee of the Capital Region of Denmark, approval numbers H-20028627 and H-20047597. The Regional Ethical Committee of the Capital Region of Denmark exempted a requirement of individual informed consent for acute infected COVID-19 patients and anonymous blood donors in leftover blood samples. Convalescent participants provided informed written consent after oral and written information.

## Conflicts of Interest Statement

The authors have no conflicts of interest to declare.

## Funding Sources

This study was supported by funds provided to Peter Garred by the EU HORIZON 2020 MSCA ITN project CORVOS 860044.

## Author Contributions

Maximilian Peter Götz, Anne Rosbjerg, and Peter Garred contributed to the study conception and design. Maximilian Peter Götz, Mikkel-Ole Skjoedt, Rafael Bayarri-Olmos, Cecilie Bo Hansen, Laura Pérez-Alós, Ida Jarlhelt, Thomas Benfield, Anne Rosbjerg, and Peter Garred commented on the manuscript; performed material preparation, data collection, and analysis; and read and approved the final manuscript. The first draft of the manuscript was written by Maximilian Peter Götz.

## Data Availability Statement

All data generated or analyzed during the findings of this study are available upon request to the corresponding author.

## Supplementary Material

Supplementary data

## Figures and Tables

**Table 1:** Descriptive statistics on the cohort of convalescent individuals

	Asymptomatic and N/A (*n* = 11)	Mild (*n* = 98)	Moderate (*n* = 171)	Severe (*n* = 61)	Critical (*n* = 6)	All (*n* = 347)
Age, years median (IQR)	67 (55–74)	47 (34–58)	52 (42–60)	64 (55–71)	59 (51–64)	52 (42–63)
Female, *n* (%)	5 (45.5)	59 (60.2)	110 (64.3)	22 (34.4)	2 (33.3)	199 (57.3)
MASP-2, ng/mL, mean (95% CI)	628.1 (416.2–840)	467.8 (424.7–511)	510 (480.2–539.9)	504.3 (453.4–555.3)	473.3 (102.6–844.1)	500.2 (477.9–522.5)

Age denoted as median (interquartile range), gender denoted as number (% of the respective population), MASP-2 concentrations denoted as mean (95% confidence interval upper and lower limit of the mean).

**Table 2:** Characteristics of hospitalized patients with moderate to severe COVID-19 according to their 30-day outcome

	Nonsurvivors (*N* = 46)	Survivors (*N* = 101)	All (*N* = 147)
Age, years, median (IQR)	81 (72–84)	71 (56–78)	74 (63–82)
Female, *n* (%)	19 (41.3)	46 (45.5)	65 (44.2)
MASP-2, ng/mL, mean (95% CI)	733.7 (620.6–846.8)	878.3 (793–963.6)	834 (765.3–902.7)

Age denoted as median (interquartile range), gender denoted as number (% of the respective population), MASP-2 concentrations denoted as mean (95% confidence interval upper and lower limit of the mean).

**Table 3:** Correlation between MASP-2 and lectin pathway PRMs in hospitalized patients with moderate to severe COVID-19

Factor	Spearman *r* (*p*)	*p* value	Significance of correlation
Ficolin-1	–0.04168	0.6162	ns
Ficolin-2	0.2906	0.0004	* **
Ficolin-3	0.3952	<0.0001	****
MBL	0.1214	0.1429	ns
CL-11	–0.08922	0.2825	ns
TCC	0.3596	<0.0001	****
CRP	0.3292	0.0002	***

CRP, C-reactive protein. Spearman correlation (ρ) calculated on nonlogarithmically transformed values. ns, *p* < 0.05;

* *p* ≤ 0.05;

** *p* ≤ 0.01;

*** *p* ≤ 0.001;

**** *p* ≤ 0.0001.
