# Supplementary material for: Lectin Pathway Enzyme MASP-2 and Downstream Complement Activation in COVID-19
Source: J Innate Immun. 2022 Jul 11;15(1):122–35. doi: 10.1159/000525508 (PMC10643890; doi:10.1159/000525508)
Supplement: Supplementary file 1 — Supplementary data [file jin-0015-0122-s01.docx]

**Lectin pathway enzyme MASP-2 and downstream complement activation in COVID-19**

**Supplementary Material**

**Materials and Methods**

**Purification of Recombinant Protein**

His-tagged rMASP-2 was purified HisPur^TM^ Ni-NTA Resin (Thermo Fisher Scientific). 500 µl of beads in equilibration buffer (PBS, pH 7.4) were added to 50 ml of rMASP-2 supernatant and incubated for 2 h end-over-end shaking. Afterward, beads were collected and washed with approx. 10 ml of wash buffer 1 (PBS, 10 mM imidazole, pH 7.4) and subsequently 10 ml of wash buffer 2 (PBS, 25 mM imidazole, pH 7.4). When washed, rMASP-2 could be eluted from the beads using approx. 10 ml of elution buffer (PBS, 250 mM imidazole, pH 7.4) while collecting the elution into 1 ml fractions. Fractions containing rMASP-2, verified by the established in-house sandwich ELISA, were collected, pooled, and finally dialyzed against HEPES buffer (25 mM HEPES (Sigma-Aldrich), 155 mM NaCl, 6.6 mM CaCl_2_). Aliquots were stored at -20°C.

**Activation of rMASP-2**

In-house produced zymogen rMASP-2 was activated as shown by Nan et al. [1,2] by adding 1 or 10 µl of TrypLE^TM^ (Thermo Fisher Scientific) to 10 µl of purified rMASP-2 (approx. stock concentration of 75 µg/ml, measured with a Qubit^TM^ Protein Quantification Assay Kit (Thermo Fischer Scientific)). After incubation for 210 s at 37°C, an amount of corn trypsin inhibitor (Prolytix, Essex Junction, VT, USA) equal to trypsin was added and the sample was placed on ice. Trypsin generally cleaves proteins at specific sites, namely between arginine or lysine and the neighboring amino acid if it is not proline [3,4].

**Detection of Activated rMASP-2**

Partial activation of rMASP-2 was verified via SDS-PAGE and subsequent Coomassie staining. Activated samples were measured on the established in-house sandwich MASP-2 ELISA. Activated rMASP-2 by trypsin was compared to zymogen rMASP-2 without trypsin after incubation at 37°C for 210 s.

**Results**

**Assay Validation: Spike-In/Recovery**

As seen in Supplementary Figure 1, dilution curves of the calibrator EDTA pool with and without spiked-in rMASP-2 supernatant follow the same slope and plateau as rMASP-2 supernatant alone. Recovery of spiked-in rMASP2 was calculated to be above 95%.

Supplementary Figure 1: Dilution curves of baseline EDTA plasma and recombinant MASP-2 culture supernatant samples, as well as spiked-in EDTA plasma samples with rMASP-2, on which recovery of MASP-2 was calculated by comparing interpolated with calculated concentrations of spiked-in samples.

A Coomassie staining of purified rMASP-2, reduced and non-reduced, activated and non-activated with trypsin, can be seen in Supplementary Figure 2. Non-activated rMASP-2 is to be seen as a single band at around 75 kDa in both conditions. Once trypsin is added, activation and cleavage products can be seen, the most important of which are the two bands in reduced conditions most probably belonging to the heavy and light chain of MASP-2 (marked with arrows) [1,2,5,6]. As the activated parts of MASP-2 are connected via a disulfide bond, the heavy and light chain will only become visible in reducing conditions when MASP-2 is activated. Other bands most probably constitute for several possible cleavage products of MASP-2 with trypsin present. These bands, showing at least partial activation of rMASP-2, can be seen to intensify with a higher concentration of trypsin present, but are also visible in the theoretically non-activated rMASP-2 sample.


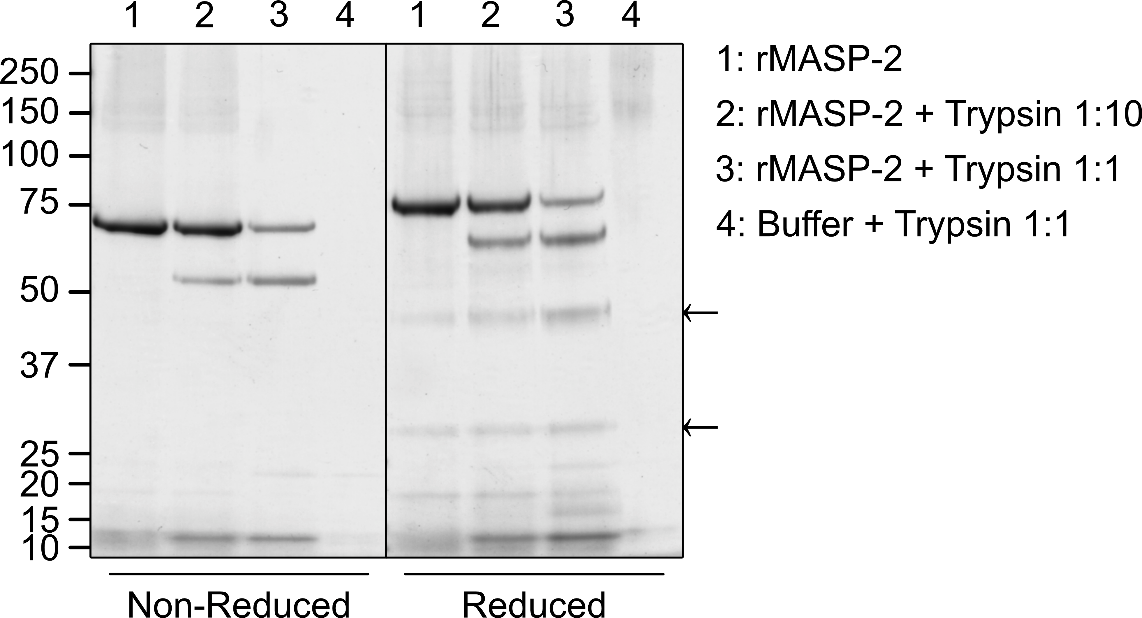


Supplementary Figure 2: Coomassie staining of zymogen rMASP-2 with and without activation by trypsin. Samples are shown after a reducing and non-reducing SDS-PAGE. The volume of trypsin was added in relation to rMASP-2. All samples were incubated for 210 s at 37°C. To every sample containing trypsin, trypsin inhibitor was added in equal amounts. As a control, no rMASP-2 was added to the last sample.

The same samples that were used for a Coomassie staining in Supplementary Figure 2 were then measured on the established in-house sandwich ELISA seen in Supplementary Figure 3. All samples in identical dilutions can be seen in overlapping curves. rMASP-2 concentrations were then interpolated and compared to the non-activated sample. The concentration of activated samples was not significantly different from non-activated samples and shared an almost identical interpolated concentration, regardless of activation status.

Supplementary Figure 3: Dilution curves of rMASP-2 with and without trypsin to activate its zymogen form. Samples were measured on the established sandwich ELISA after incubation at 37°C for 210 s with and without trypsin. To every sample containing trypsin, an equal amount of trypsin inhibitor was added.

**References**

1 Nan R, Furze CM, Wright DW, Gor J, Wallis R, Perkins SJ. Flexibility in Mannan-Binding Lectin-Associated Serine Proteases-1 and -2 Provides Insight on Lectin Pathway Activation. Structure. 2017 Feb;25(2):364–75.

2 Chen C-B, Wallis R. Two Mechanisms for Mannose-binding Protein Modulation of the Activity of Its Associated Serine Proteases*. J Biol Chem. 2004;279(25):26058–65.

3 Simpson RJ. Fragmentation of protein using trypsin. CSH Protoc. 2006 Oct;2006(5). DOI: 10.1101/pdb.prot4550

4 Keil PDB. Specificity of Proteolysis. Springer Berlin Heidelberg. 1992.

5 Gulla KC, Gupta K, Krarup A, Gal P, Schwaeble WJ, Sim RB, et al. Activation of mannan-binding lectin-associated serine proteases leads to generation of a fibrin clot. Immunology. 2010 Apr;129(4):482–95.

6 Thiel S, Kolev M, Degn S, Steffensen R, Hansen AG, Ruseva M, et al. Polymorphisms in Mannan-Binding Lectin (MBL)-Associated Serine Protease 2 Affect Stability, Binding to MBL, and Enzymatic Activity. J Immunol. 2009;182(5):2939–47.
